# Curcumin Resource Database

**DOI:** 10.1093/database/bav070

**Published:** 2015-07-25

**Authors:** Anil Kumar, Hasnahana Chetia, Swagata Sharma, Debajyoti Kabiraj, Narayan Chandra Talukdar, Utpal Bora

**Affiliations:** ^1^Bioengineering Research Laboratory, Department of Biosciences and Bioengineering, Indian Institute of Technology Guwahati (IITG), Assam 781039, India,; ^2^Centre for Biological Sciences (Bioinformatics), Central University of South Bihar (CUSB), Patna 800014, India,; ^3^Institute of Advanced Studies on Science and Technology (IASST) Boragaon, Guwahati, Assam 781035, India and; ^4^Institutional Biotech Hub, Centre for the Environment, Indian Institute of Technology Guwahati (IITG), Assam 781039, India

## Abstract

Curcumin is one of the most intensively studied diarylheptanoid, *Curcuma longa* being its principal producer. This apart, a class of promising curcumin analogs has been generated in laboratories, aptly named as Curcuminoids which are showing huge potential in the fields of medicine, food technology, etc. The lack of a universal source of data on curcumin as well as curcuminoids has been felt by the curcumin research community for long. Hence, in an attempt to address this stumbling block, we have developed Curcumin Resource Database (CRDB) that aims to perform as a gateway-cum-repository to access all relevant data and related information on curcumin and its analogs. Currently, this database encompasses 1186 curcumin analogs, 195 molecular targets, 9075 peer reviewed publications, 489 patents and 176 varieties of *C. longa* obtained by extensive data mining and careful curation from numerous sources. Each data entry is identified by a unique CRDB ID (identifier). Furnished with a user-friendly web interface and in-built search engine, CRDB provides well-curated and cross-referenced information that are hyperlinked with external sources. CRDB is expected to be highly useful to the researchers working on structure as well as ligand-based molecular design of curcumin analogs.

**Database URL:** http://www.crdb.in

## Introduction

Curcumin (diferuloylmethane) is a hydrophobic polyphenol derived from rhizome of the perennial herb turmeric (*Curcuma longa*) which belongs to the ginger family (Zingiberaceae) native to tropical South Asia (1). Numerous traditional usage of turmeric is described in Indian and Chinese traditional medicine for prevention and treatment of a broad range of diseases and conditions (2, 3). These uses include turmeric pastes for treating eye infections, burns and bruises; turmeric powder with milk for respiratory ailments; and roasted turmeric for dental and digestive disorders (4, 5). Its traditional utility was limited not only to therapeutics but also to textile and cosmetic industries (5). With the advent of western medicine, the use of turmeric for medication went into oblivion for a long time. However, record of more than 13,000 scientific articles published during the last three decades suggests that there is a resurgence of interest in turmeric and its principal therapeutic component, curcumin.

**Figure 1.**
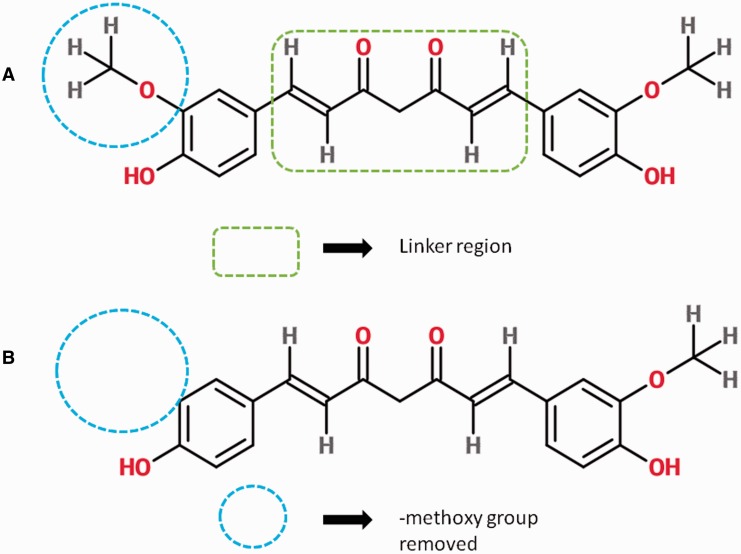
The basic structure of Curcumin (A) consists of a linker region connecting two aromatic rings. Chemical modifications such as removal of methoxy group from one aromatic ring give rise to a new curcumin analog, in this case, Demethoxycurcumin (CRDB_AN000434) (B).

Current research in curcumin and its analogs (curcuminoids) is focused on exploring their potential use as antimicrobial, antioxidant, anti-inflammatory, onco- preventive, neuroregenerative agents and other health benefits (6–9). Curcumin has been found effective in decreasing serum cholesterol and attenuation of cardio-toxicity (10). Curcumin has also shown a significant role in treatment of neurological disorders such as Alzheimer’s disease, Parkinson’s disease and anxiety because it acts on oxidative stress and inflammation-related responses in these diseases that share similar biochemistry (11, 12). Similarly, it can also inhibit autoimmune diseases by regulating cytokines such as IL-12 and IL-6 (13). Curcuminoids have also been reported to interact with a huge number of molecular targets such as signaling molecules, transcription factors, growth factors, receptors, adhesion molecules, pro-inflammatory enzymes and protein kinases (2, 4, 14). Many synthetic analogs have been designed by adding various functional groups in aromatic rings and linker region as shown in Figure 1 (15). These analogs have shown improved activity relative to curcumin (16). Curcumin–amino acid and curcumin–oligonucleotide conjugates have also been synthesized and tested for several pharmacological properties (17, 18).

With the increase in information on curcumin available from different sources, it is becoming essential to build a single platform to store these data along with their interrelations. Curcumin Resource Database (CRDB v1.1) has been created to host these diverse arrays of information regarding curcumin and its analogs by combining different data sources. It is expected that this database will act as a standalone information resource for researchers and common people.

## Data content and sourcing

**Figure 2.**
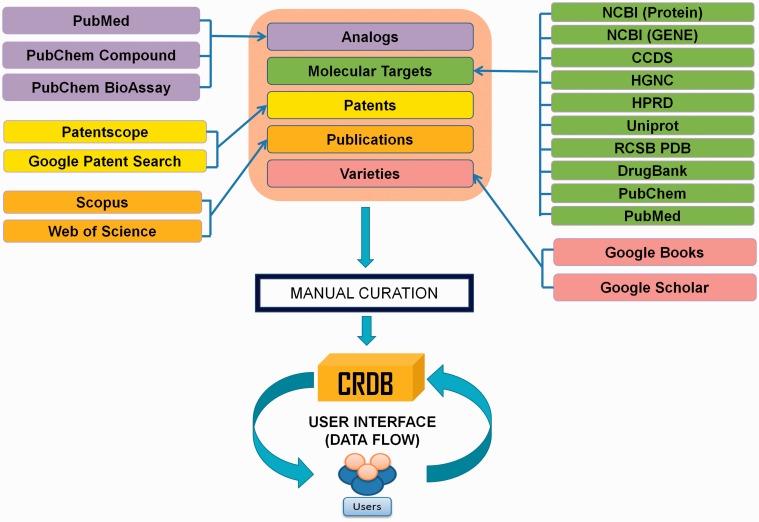
Workflow for the construction of the CRDB v1.1.

In CRDB v1.1, the dataset have been organized in five different modules, namely ‘Curcumin Analogs’, ‘Molecular Targets of Curcumin’, ‘Curcumin-based Patents’, ‘*Curcuma longa* Varieties’ and ‘Publications on Curcumin’. The workflow along with sources adopted for construction of CRDB has been schematically shown in Figure 2. An easy-to-use web interface equipped with an in-built search engine as well as an exclusive download link allows a remote user to retrieve and contribute data to CRDB without hassle.

## Database design and implementation

The design of CRDB consists of the following features and functionalities:

- Least site opening time.
- Clean and professional design.
- User registration modules.
- Homepage and child pages.
- Sitemap, content pages.
- Dynamic webpage.
- Search filter mechanism.
- Search engine optimization friendly.
- Compatibility with all browsers.
- Visitor counter in footer area.
- Other links in side bar.
- Frequently asked questions.
- Feedback mechanism.

**Figure 3.**
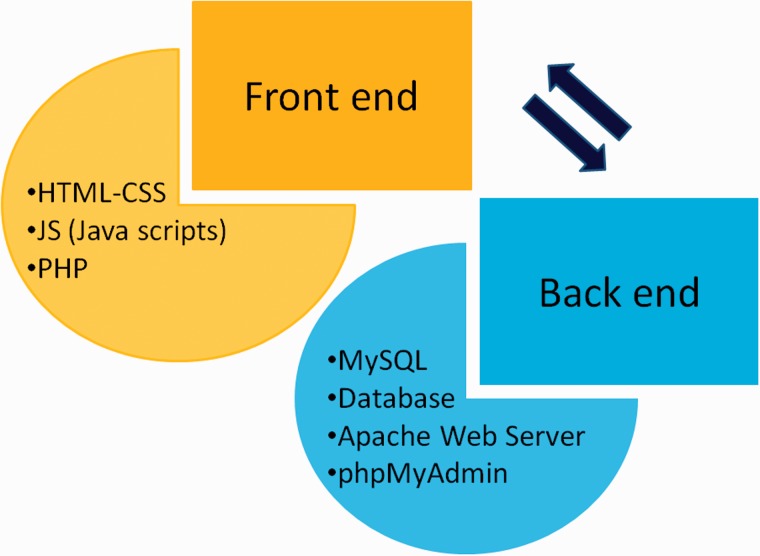
Web development strategy of CRDB.

**Figure 4.**
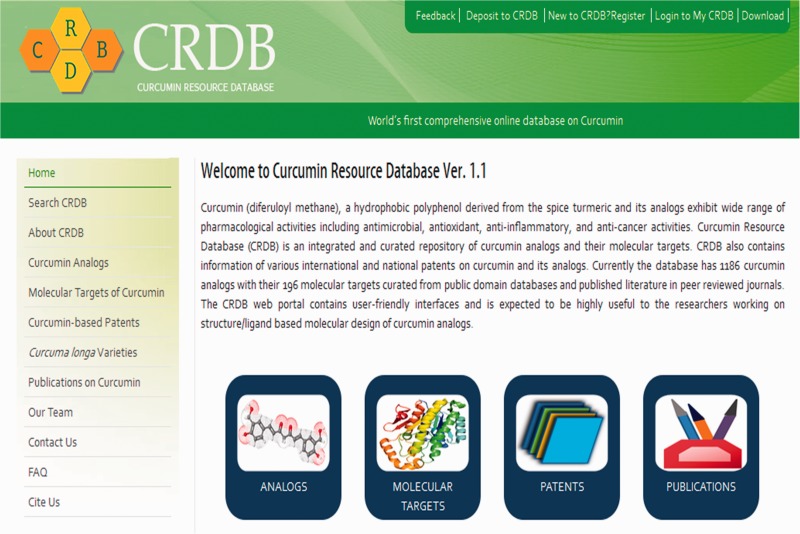
Web interface of CRDB.

**Figure 5.**
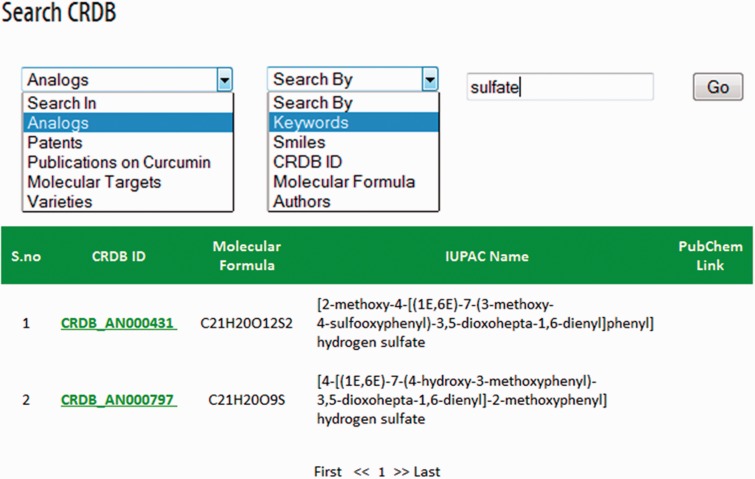
CRDB search engine.

CRDB is implemented in a PHP (Hypertext Preprocessor) module with Java scripts running in the front end whereas MySQL database and Apache web server at the back end as shown in Figure 3. A PHP security system was included in CRDB to prevent data infringement and data hack. This architecture resides upon an Intel Pentium 4 processor and running on Linux-based server. The web interface comprises a collection of web pages related to the data provided as shown in Figure 4. The search page of the database serves as the gateway to query the database contents dynamically as instructed by the user through various options (dropdown menus/keyword search) as shown in Figure 5. The data collected from different sources are initially stored in manually curated Microsoft Excel spreadsheets, saved in .csv (comma separated values) format and uploaded to the database server. The database works well with commonly available web browsers, such as Mozilla Firefox, Google Chrome and Microsoft Internet Explorer.

## Construction and curation of data content

For collection of raw data, ‘curcumin’ was used as a keyword in search bars of various public domain databases. For example, to obtain the analogs of curcumin, the keyword ‘curcumin’ was searched in PubChem Compound (19). Then, we searched for ‘similar compounds’ of the first hit from the search results. The output of this operation gave rise to around 1300 curcumin-like compounds out of which the ones that shared the similar backbone structure of curcumin (refer to Figure 1A) were retained. After curation, the ‘bioassays’ listed in PubChem for the analogs were screened to obtain the corresponding molecular targets. Similarly, protein sequences were extracted from NCBI Protein, two-dimensional macromolecular structures from PubChem Compound, publications on curcumin from Scopus (http://www.sco pus.com/) as well as Web of Science (http://apps.webof knowledge.com), patent data from PATENTSCOPE (https://patentscope.wipo.int/search/en/search.jsf), etc. (20). Other relevant information was acquired from DrugBank, Uniprot, HGNC (HUGO Gene Nomenclature Committee), HPRD (Human Protein Reference Database), RCSB PDB (Protein Database), etc. (21–25).

At present, the database has 1186 curcumin analogs with their 195 molecular targets, 489 patents, 176 *C.* *longa* varieties and 9075 peer-reviewed publications. All the entries are hyperlinked with their respective source databases.

A unique CRDB identifier (ID) has been created for each entry with a common prefix ‘CRDB_’ and suffix ‘AN’ for analogs, ‘MT’ for molecular targets, ‘PT’ for patents, ‘VR’ for varieties and ‘LT’ for publications.

## Data access and query options

CRDB can be accessed at http://www.crdb.in. This database can be browsed via the respective pages of analogs, molecular targets, patents, publications and varieties. Search bars have been incorporated in each page; however, a separate webpage for search has also been provided for flexible navigation. In common, search can be carried out using CRDB ID (a database-specific identifier for each data entry) and keywords. Other options of search vary among different web pages. For instance, search options for ‘Curcumin Analog’ page are keywords, smiles, molecular formula, CRDB ID and authors. Similarly, those for ‘Publications on Curcumin’ page are CRDB ID, keywords, year and author.

## User registration and feedback

A user can register in CRDB using the ‘Register’ tab using their e-mail ids. This enables them to submit as well as download data from the database. Similarly, a user can provide feedback to the database using the feedback form.

## Data submission, update and retrieval

There is a provision to submit new data in CRDB under ‘Deposit to CRDB’ tab. A user has to register in CRDB prior to data submission. This step is implemented to keep track of users providing genuine as well as false data. Post-login, a user can submit their data using interactive data entry forms. Once the data are submitted, the admin will first verify the data from the references cited. Additionally, admin will check for duplicate entries in the CRDB. Once no anomaly is found, the entry will be allotted CRDB ID and the information will become available for public viewing. Apart from addition of user submitted data, the web interface of CRDB v1.1 will be updated annually to add new category of data and novel features. To download the complete dataset of each data type in spreadsheets (.csv format) from the ‘Download’ tab, the user has to similarly register with CRDB.

## Database utility

Most of the information provided by CRDB can also be obtained from other databases. But the information are scattered with no interlinks among them. In contrast, CRDB acts as the network encompassing all these data and interconnecting them.

CRDB has the potential to assist in multi-dimensional applications such as high-throughput screening and target-based drug design. Knowledge of curcumin content in different varieties of *C.* *longa* can have an impact on agriculture, food-based as well as pharma-based industries. Information on Patents and publications provide an added perk for the users, helping them in literature review as well as avoidance of repetitive research and innovation.

## Future developments in CRDB

At present, CRDB represents one of the most data- intensive repositories for curcumin. In future, we are planning to include various drug delivery systems for curcumin as well as traditional uses of curcumin. To synchronize future updates of CRDB with the present one, we have introduced a versioning system and references will be available for different versions.
